# Recommendations on dose level selection for repeat dose toxicity studies

**DOI:** 10.1007/s00204-022-03293-3

**Published:** 2022-04-29

**Authors:** Fiona Sewell, Marco Corvaro, Amanda Andrus, Jonathan Burke, George Daston, Bryan Delaney, Jeanne Domoradzki, Carole Forlini, Maia Louise Green, Thomas Hofmann, Sven Jäckel, Moung Sook Lee, Michael Temerowski, Paul Whalley, Richard Lewis

**Affiliations:** 1 National Centre for the Replacement, Refinement and Reduction of Animals in Research (NC3Rs), Gibbs Building, 215 Euston Road, London, NW1 2BE UK; 2 Corteva Agriscience Italia S.R.L., Rome, Italy; 3 The Dow Chemical Company, Midland, MI USA; 4 Reckitt, Hull, UK; 5 The Procter and Gamble Company, Cincinnati, OH USA; 6 Firmenich, Plainsboro, NJ USA; 7 Corteva Agriscience, Indianapolis, IN USA; 8 Arkema, Colombes, France; 9 ExxonMobil Biomedical Sciences Inc., Annandale, NJ USA; 10 BASF SE, Ludwigshafen, Germany; 11 Merck KGaA, Darmstadt, Germany; 12 Clariant, Sulzbach am Taunus, Germany; 13 Bayer Crop Sciences, Mannheim, Germany; 14 Syngenta Ltd., Bracknell, UK

**Keywords:** Dose selection, Toxicokinetics, Exposure, 3Rs

## Abstract

Prior to registering and marketing any new pharmaceutical, (agro)chemical or food ingredient product manufacturers must, by law, generate data to ensure human safety. Safety testing requirements vary depending on sector, but generally repeat-dose testing in animals form the basis for human health risk assessments. Dose level selection is an important consideration when designing such studies, to ensure that exposure levels that lead to relevant hazards are identified. Advice on dose level selection is provided in test guidelines and allied guidance documents, but it is not well harmonised, particularly for selection of the highest dose tested. This paper further builds on concepts developed in a technical report by the European Centre for Ecotoxicology and Toxicology of Chemicals (ECETOC) which recommends pragmatic approaches to dose selection considering regulatory requirements, animal welfare and state of the art scientific approaches. Industry sectors have differing degrees of freedom to operate regarding dose level selection, depending on the purpose of the studies and the regulatory requirements/legislation, and this is reflected in the overall recommended approaches. An understanding of systemic exposure should be utilised where possible (e.g., through toxicokinetic approaches) and used together with apical endpoints from existing toxicity studies to guide more appropriate dose level selection. The highest dose should be limited to a reasonable level, causing minimal but evident toxicity to the test animals without significantly compromising their well-being. As the science of predictive human exposure further develops and matures, this will provide exciting and novel opportunities for more human-relevant approaches to dose level selection.

## Introduction

Human health toxicologists are well equipped to describe and/or predict the potential adverse effects that a chemical may cause to humans. Current methods to investigate the effects of prolonged chemical exposures rely heavily on repeated dose toxicity studies conducted in animals and the identification of hazards. Though study designs and approaches to dose level selection have evolved over time, these continue to focus on describing adverse (hazardous) effects, be these at the genetic, cellular, organ or whole organism level. However, it is important not to focus solely on identifying and characterising a hazard—these need to be put into context. With the goal of ensuring human safety, the *relevance* of observed hazards and, therefore, risk to humans needs to be understood, and this requires consideration of use context and human exposure, something that can be assessed more easily for certain industry sectors (such as pharmaceuticals or agrochemicals) but much more difficult to accurately assess for other types of chemicals. Moreover, since in vivo toxicity studies generally rely on only a small number of dose levels (usually no more than four), it can be difficult to establish dose–response relationships to accurately describe the exposure levels (doses) that cause (or can be predicted to cause) adverse effects. It is, therefore, critical that appropriate dose levels are selected to provide the optimum data but also pragmatic information to serve the goal of protecting human health, rather than academic curiosity.

An additional challenge faced globally is the different ways in which information from toxicity studies is used in risk management. For risk assessment, the critical data from repeat dose toxicities studies are the No Observed Adverse Effect Level (NOAEL) or the derived benchmark dose which are used as the point of departure (PoD) from normality (e.g., derived no effect levels (DNELs) and predicted no effect concentrations (PNECs)). This is compared to estimated or predicted human exposure to provide a risk assessment, to subsequently allow a risk management judgment to be made on human safety for that chemical application or exposure. When data are used for hazard-based classification the focus is purely on the effect (hazard), independent of toxicological potency and relevance to human exposure. This means that hazard-based classification is a rather blunt instrument for protecting human health as there is little consideration of the degree of hazard or the relevance of the dose levels used in toxicity studies to human exposures. This publication on dose level selection is timely in view of wider activities in the area of chemical regulation. The EU Chemicals Strategy for Sustainability (EU 2020), has attracted significant comment and criticism from the regulatory and scientific communities for its focus on hazard and for its intent to further regulate non-existent or well considered risks (Herzler et al 2021; Barile et al. 2021). The importance of the weight of evidence approach described in this publication is even more relevant given the increasing dichotomy and polarisation of approaches to dose level selection for some industry sectors as exemplified in recent guidance from the European Chemicals Agency (ECHA 2021, 2022a and b).

This paper builds on (and illustrates through further examples), concepts developed in the extensive review, analysis and recommendations of the European Centre for Ecotoxicology and Toxicology of Chemicals (ECETOC; ECETOC Technical Report 138, 2021) and is intended to recommend sector-specific approaches that protect human health while considering animal welfare, providing relevant endpoints for risk assessment and the information needed for hazard-based classification. Workable approaches that allow risk assessment and hazard-based classification to co-exist are a priority.

## Current guidance

Current guidance on dose level selection is largely based on advice within the Organisation for Economic Co-operation and Development (OECD) Test Guidelines (TG), a library of internationally agreed testing methods used by government, industry and independent laboratories to identify and characterise potential hazards of chemicals. The original aim of the OECD TGs and associated Guidance Documents was to produce a well-defined framework for each toxicity test to standardise conduct so that results are fully acceptable to numerous regulatory agencies (under the so-called Mutual Acceptance of Data). This harmonised approach aimed to (i) promote scientific aspects of toxicity testing, (ii) ensure international acceptance of test data, (iii) avoid test duplication, (iv) promote efficient use of laboratory animals and (v) improve the efficiency of test conduct. The guidelines were intended to align general principles of toxicity testing to detect and characterise hazard in a reproducible manner. They were not specifically designed for uses such as hazard classification and labelling purposes for which the data are particularly used today, and their use in this context should be treated with pragmatism and caution. The guidelines were designed to propose methodologies that could be used across chemical sectors including agrochemical, biocides, food ingredients, consumer products, industrial chemicals and pharmaceuticals (although the latter now supplanted by the guidelines produced by the International Council for Harmonisation of Technical Requirements for Pharmaceuticals for Human Use (ICH)) to facilitate risk assessment. These were *not* intended to be a stringent test protocol, but to instead lay out a set of generally accepted principles that could be modified for each industry sector or purpose and to provide comparable endpoints. However, their use may now be viewed as more prescriptive especially around the guidance provided on dose level selection.

Although OECD TGs are subject to periodic revision and additions, the fundamental core of most TGs has generally remained unchanged since their development over 40 years ago. The results and endpoints derived from these studies are still being used within the regulatory arena today, both to identify and characterize hazards, as well as for risk assessment. As for any test involving animals, there are limitations, and it is recognised that the animal models used in repeat dose studies may not always be relevant when extrapolating to humans. Prior to conducting each toxicity test, important considerations include the final purpose of the study and the dose relevance for the use pattern and exposure scenario for the chemical of interest. For example, is the focus on risk assessment or on hazard characterisation? What is the probability (risk) of a human ever encountering repeat chronic exposures anywhere near the current limit dose of 1000 mg/kg body weight (bw)/day for repeat dose toxicity studies (i.e., 60 – 75 g per day for an average adult human being)? The current ‘top down’ approach to dose level selection, starting with an excessively high dose, may not be fit-for-purpose and instead a ‘bottom up’ approach, using a knowledge or prediction of human exposure, to inform dose ranges may be more relevant (Sewell et al. 2021).

As mentioned, OECD TGs are not intended to be a test protocol, and they do not contain the level of detail set out in Standard Operating Procedures (SOPs), including on approaches to and rationale for dose level selection, and, therefore, toxicological skill and pragmatic judgment are needed to ensure appropriate dose level selection. It is important to note that current OECD TGs are generally hazard focused, with the stated purpose being to identify and characterise a particular hazard/ endpoint to provide a PoD for a risk assessment. This is demonstrated in advice found on dose level selection and study purpose outlined in individual test guidelines, as follows:

- *Study purpose* The following excerpt taken from OECD 408, 2018 (90 day Repeated Oral Toxicity study in Rodents), summarises the test purpose and is typical of the guidance given for repeat dose studies: *“….The study will provide information on the major toxic effects, indicate target organs and the possibility of accumulation of test chemical, and can provide an estimate of a NOAEL of exposure which can be used in selecting dose levels for chronic studies and for establishing safety criteria for human exposure. Alternatively, this study yields dose related response data that may be used to estimate point of departure for hazard assessment using appropriate modelling methods (e.g., benchmark dose analysis).”*
- *Dose level selection (general)* Taking this same guideline as an example the advice on dose level selection has a focus on hazard characterisation, identifying a PoD for use in risk assessment, and advocating a top-down approach: *“…Dose levels may be based on the results of repeated dose or range finding studies and should take into account any existing toxicological and toxicokinetic data available for the test compound or related materials. A descending sequence of dose levels should be selected with a view to demonstrating any dosage related response and a NOAEL at the lowest dose level…”*
- *Dose level selection (chronic studies)*. Further but minimal advice on dose level selection is given in Guidance Document 116 (OECD 2014), (in the context of long term and carcinogenicity studies). However, this advice is far from definitive and even notes controversy: *“…Dose selection should be based on the findings of subchronic or range-finding studies…Ideally, the dose levels selected will maximise the detection of dose–response relationships and facilitate the extrapolation of these to potential hazards for other species, including humans. The selection of the highest dose level to be used in a chronic toxicity or carcinogenicity study has long been a matter of controversy. At the time when long-term animal bioassays began to be routinely used to assess the qualitative potential of a test substance to cause chronic toxicity and cancer, the emphasis was on testing at high levels to maximise the potential of such studies to detect effects…”*
- *Maximum tolerated dose (MTD)*. This focus on hazard identification gave rise to the ‘MTD’ (OECD, 2014). *“…The concept of the Maximum Tolerated Dose, conventionally defined as the highest dose to produce toxic effects without causing death and to decrease body weight gain by no more than 10% relative to controls (*OECD 2002a*—GD No. 35) became well established. The MTD is often used in the assessment of a chronic toxicity or a carcinogenicity study to decide whether the top dose tested was adequate to give confidence in a negative result. …While some regulatory bodies or organisations interpret an adequate high dose to be a minimally toxic dose, others emphasise the need to select a dose level that is a maximally tolerated dose (i.e., more severe toxicity should be demonstrated). Thus, because of differences in views regarding the severity of toxic effects that are interpreted as providing evidence that an adequate high dose has been attained or exceeded, a completed carcinogenicity bioassay may be considered to be acceptable by one organisation but not by another….There is broad acceptance that the top dose should ideally provide some signs of toxicity such as slight depression of body weight gain (not more than 10%), without causing e.g., tissue necrosis or metabolic saturation and without substantially altering normal life span due to effects other than tumours. Excessive toxicity at the top dose level (or any other dose level) may compromise the usefulness of the study and/or quality of data generated…"*

As for any tests involving experimental animals it is important to ensure a humane approach is used, including avoiding the use of excessively high doses that cause harm to animals. The scientific benefits of a study and harm to the animals used need to be balanced (see section on ethical considerations below). Particularly with respect to the animals’ well-being, it needs to be taken into account that dose selection nearly always involves a prediction of what might happen in a study of longer duration than available in the current data base (e.g., dose selection for a 90-day exposure period based on information from a 28-day study). The ECETOC Technical Report 138 (2021) contains more detail on the evolution of concepts such as the MTD and how they can be used within different contexts and study durations. As mentioned above, OECD TGs have evolved over decades and we have taken the opportunity to review and make recommendations on best practice.

## Meeting the needs of chemical classification

The purpose of chemical regulation is to reduce and minimize ill health (harms) caused by exposure to chemicals. To achieve this, the potential for a chemical to cause harm needs to be defined and compared with expected human exposure (either measured or estimated). Irrespective of the approach used (hazard or risk-based), the hazard should be understood sufficiently to allow implementation of risk management decisions to mitigate/minimise potential adverse effects on public health. The hazard should, therefore, be defined in terms of the effect produced and the exposure required *to cause* that effect. However, this isn’t always the case, for example with the Globally Harmonised System (GHS; United Nations 2021).

The GHS, a single worldwide system for classifying and communicating the hazardous properties of industrial and consumer chemicals, is well established and internationally agreed. It was designed to ensure the consistent and standardised classification of chemical hazard globally, though there are also regional adaptations of this guidance (for example for Classification Labelling and Packaging (CLP) in the EU according to Regulation EC No 1272/2008; EU 2021). Guidance in the latest GHS document on classification and labelling (9th revision ed, 2021), states that when a specific classifiable adverse event or specific target organ toxicity is identified from standard well-conducted studies, then it should be evaluated and classified and when necessary placed in a specific category. Some categories are associated with a specific need to provide a label on the safety data sheet or container, to notify relevant personnel when transporting and to provide advice on handling and on protective equipment.

Hazard-based approaches to classification such as the GHS system use a weight of evidence approach to determine if a chemical has the potential to cause a particular type of adverse effect. Although the severity of an effect is taken into account for key classifications such as those for carcinogenicity, mutagenicity and reproductive toxicity (CMR) the potency of a chemical that may cause these adverse effects is not considered. This means that all chemicals deemed to be hazardous for a given effect are subject to the same risk management decisions irrespective of potency. There is no element of risk assessment in this process. These decisions can in some circumstances (e.g., for some sectors, endpoints and/or regions) lead to binary outcomes (i.e., classified/not classified) which, in the presence of established hazard-based cut-off criteria, can lead to ban or restrictions of use of a certain chemical based on hazard alone.

Whilst systems such as the GHS exist where classifications are based on having a relevant finding in the appropriate toxicology studies, there is no requirement to arrive at a positive classification outcome for every molecule. In many cases (and especially for the key classifications related to mutagenicity, cancer and reproduction), evaluation of the hazard data shows that classification is *not* required as the chemical does not cause these adverse effects. Complex endpoints such as mutagenicity, cancer and reproduction may be influenced by other factors unrelated, or only partly related, to the intrinsic hazardous property of interest, including dose level selection, duration of treatment, corrosivity at site of contact, species and strain of the test model and may not be directly attributable to the systemic toxicity of the molecule in question. To consider these as an intrinsic feature of the chemical (i.e., an intrinsic hazard) may be considered a flawed premise. While it is true that some acute toxicities such as corrosivity may be intrinsic to the molecular structure it is equally true that carcinogenicity and reproductive toxicity are not intrinsic features of the molecule. This is because the development of cancer and adverse effects on reproduction, for example, are complex multistage processes where each stage (or key event) has its own dose response and temporal relationship.

Recently, concerns have been expressed that insufficient dosing in some toxicity studies (particularly those relating to assessments of reproductive toxicity, mutagenicity and cancer) may provide inadequate data for classification and labelling purposes (Heringa et al. 2020; Woutersen et al. 2020). The over-riding concern being that by missing elements of hazard it may not be possible to fulfil the precautionary protection goal served by classification and labelling. Whilst these concerns are driven by a wish to ensure adequate human health protection (albeit from a hazard-based perspective), this approach is highly likely to be overly conservative and there are concerns that it could lead to the use of unnecessarily high doses in animal studies that do not add scientific value (Sewell et al. 2020; Smith and Perfetti, 2020; Terry et al. 2020). As ‘the dose makes the poison’, most chemicals will show an adverse effect if dosed at high enough levels. Dose level selection should be based on identifying a relevant hazard and providing a point of departure for risk assessment.

### Ethical considerations

It is important that the scientific and regulatory aims of a study are balanced against any potential effects seen in animals and that studies comply with the 3Rs (Replacement, Reduction and Refinement of animals in research). Developed over 60 years ago to provide a framework for performing more humane animal research (Russell and Burch 1959) these principles are now embedded into scientific practice and incorporated into the associated resources, guidance and legislation. As well as incorporation into specific guidelines, the OECD has a separate guidance document outlining a framework for the recognition, assessment and use of clinical signs as humane endpoints for experimental animals used in safety evaluation (OECD 2002b), which was heavily influenced by the work of other organisations and recognised experts, such as recommendations of the Federation for Laboratory Animals Science Associations (FELASA) (Guillen 2012). Regional legislation and guidance (e.g., European Directive 2010/63/EU) may stipulate the minimal conditions that need to be met when conducting experiments in animals, but there is a wealth of information from other recognised experts on topics such as euthanasia and recognition of pain that may be useful to ensure the appropriate implementation of humane endpoints (Hawkins et al. 2016; Leary et al. 2020).

Relevant resources include pragmatic advice on the use of mild, moderate and substantial severity signs to support selection of the MTD within regulatory general toxicology studies for pharmaceuticals (FELASA 1994; reviewed and updated in LASA/NC3Rs 2009). In the more recent version, the authors point out that *“Defining the MTD in the studies of shortest duration informs dose setting in subsequent studies and is crucially important in application of the 3Rs since this reduces the chances of larger numbers of animals that are used in regulatory studies being exposed to unanticipated suffering”.* Essentially, when a single substantial effect or a combination of moderate effects is observed (e.g., effects prolonged in nature such as a body weight loss up to 20%, or a reduction of feed consumed higher than or equal to 60% for more than 72 h), this should result in immediate actions including, where appropriate, euthanasia. While mild effects, such as reduced weight gain, transient postural, neurological, respiratory, cardiac signs and/or mild temporary reduction (25–60%) of feed consumption could be considered acceptable for short term studies, current thinking is that there is no value in exceeding this or in demonstrating moderate toxicity.

There is a continuing debate around acceptable body weight loss limits and the impact this has on study and/or humane endpoints. More recent studies have shown that, in terms of body weight loss, the previous guidance is conservative, and that toxicity should be limited to only mild clinical signs. The UK NC3Rs collected data from 151 studies from 15 organisations and proposed to reduce the body weight loss limit for short term dosing (up to 7 days) to 10% for rat and dog and 6% for non-human primates (Chapman 2013). The evidence clearly indicated that even for initial short-term repeat dose studies there is no justification for exceeding dose levels that cause only mild effects—when body weight loss exceeded the recommended limits the study always needed to be stopped or animals euthanised, unless associated with the expected pharmacology (e.g., metabolic disturbances, reduced appetite). Body weight loss as an objective indicator of MTD is supported by a similar cross-company initiative within the chemicals industry (mainly agrochemicals), where data on clinical signs observed during acute inhalation toxicity studies (single exposure followed by observation for up to 14 days duration) in rats was shared. Statistical analyses showed that body weight loss in excess of 10% compared to starting weight is highly predictive (positive predictive value of 94%) of death at higher concentrations, showing that the MTD had already been reached or exceeded (Sewell et al. 2015). At an International Life Sciences Institute (ILSI)–Health and Environmental Sciences Institute (HESI) workshop dealing with maternal toxicity (Beyer et al. 2011) there was no consensus on what would be deemed acceptable in terms of an impact on body weight gain. However, regarding developmental and reproductive toxicity studies a 20% decrease in body weight gain was considered excessive.

### The relevance of observed effects

It is recognised that certain toxicities observed in experimental animals and often at high dose levels are species specific (e.g., alpha-2u globulin accumulation causing kidney toxicity in male rats, and rodent liver growth leading to liver tumours and compensatory thyroid hyperplasia secondary to liver toxicity) and are, therefore, not relevant to humans. In some industry sectors (e.g., pharmaceuticals and food ingredients) and under certain circumstances, high dose testing in animals is often considered irrelevant in informing hazard and risk decisions for *human relevant* exposure levels. Rather than focus testing and attention on high dose phenomenon, more value is placed on the precision of dose level selection and the relevance of effects in the sub-MTD range as this can provide more relevant information on target organ toxicity.

The purpose of this guidance is to provide sector-specific recommended approaches to protect human health, respect animal welfare and provide relevant endpoints for risk assessment and the information needed to assign hazard-based classification. However, it is recognised that different sectors have differing degrees of freedom to operate in dose level selection, and this is discussed in more detail. For example, in cases where chemicals are designed to be biologically active (pharmaceuticals and agrochemicals), there is usually a large amount known about the chemical and/or mode of action class that can be used to guide dose level selection. However, in other situations (industrial chemicals and food ingredients) there may be less or even an absence of information to guide dose level selection. In addition, the different sectors are regulated in different ways and the type of information accepted for use in dose level selection across sectors varies accordingly. These differences are reflected in the individual sector recommended options and approaches (ECETOC Report 138, 2021, Table 1). Recommendations take into account the industry sector, the practices and expectations of regulators in that sector in different regions, the likely route of human exposure as well as other factors such as route specific Absorption, Distribution, Metabolism, and Excretion (ADME).

**Table 1:** Cross-sector comparison of challenges for dose selection within toxicity studies, and opportunities to use kinetic data

	Pharmaceuticals	Agrochemicals	Industrial chemicals	Food
Purpose and consequence of regulatory toxicology studies	▪To support/enable dose selection in human clinical trials and/or subsequent in vivo animal toxicology studies ▪Additional clinical monitoring or clinical holds can be placed	▪Studies used for CLP and risk assessment ▪Product can be banned based on specific hazards (e.g., carcinogen, mutagen, DART)	▪Studies used for CLP and risk assessment ▪Clarify a concern that the manufacture and/or use of the substances could pose a risk to human health or the environment ▪Product can be banned based on specific hazards (e.g., carcinogen, mutagen, DART)	▪Studies used for CLP and risk assessment ▪Product could be classified by regulatory agencies based on results observed in high dose (i.e., limit dose) toxicology studies that are not relevant for human exposure
Available guidance on dose selection	Generally harmonised and flexible e.g., ICH, but some regional differences	▪Some, but not always harmonised e.g., differences in OECD TGs, OECD guidance documents, EPA or other regional guidance	▪Some but not always harmonised, e.g., differences in OECD TGs, and EPA (or other) regional guidance	▪Limit dose normally expected unless otherwise justified
Dialogue with regulators	▪Encouraged	▪Not usual; where possible, lack of consistency in regulatory positions between and within regions	▪Not usual	▪Not common and can lack consistency in positions
Knowledge of Mechanism of Action (MOA)	▪Yes, pharmacological/off-target MOA generally known	▪Yes, biological MOA generally known, may be related or not to toxicity MoA	▪MOA rarely known	▪MOA rarely known
Challenges relating to dose selection for toxicity studies	▪Define a NOAEL or STD10/HNSTD to enable the calculation of the dose levels for the first clinical trial in human	▪Current toxicity testing paradigms often require doses used to exert toxic effects ▪Limited human data (mostly in vitro) ▪Limited PBPK models pharma-focussed; dietary, dermal, inhalatory routes of exposure ▪CLP requirements are hazard-focused	▪Current toxicity testing paradigms require that high doses are used to exert toxic effects ▪Using a relevant high dose to avoid the need to repeat the study (animal welfare, additional costs, lost time) ▪Rarely able to generate human data ▪Limited PBPK models and existing ones pharma-focussed; oral route of exposure only ▪CLP requirements are hazard-focused	▪Some current toxicity testing paradigms require doses that produce toxic effects ▪Mixtures − kinetic analysis challenging particularly for dietary exposure studies ▪Rarely able to generate human data ▪CLP requirements are hazard-focused
Approaches applied to understand exposure on toxicity studies	▪PK incorporated on to studies as standard to inform dose setting and understand internal dose ▪Microsampling approaches in use to reduce need for additional TK satellite groups ▪Refined dose-setting is accepted in general toxicity studies (e.g., ICH M3 R2: 50 ×) ▪Mathematical PBPK models routinely used to establish internal exposures, especially following oral exposure ▪TTC approach accepted for genotoxic impurities ▪QSAR approaches in use/development	▪Kinetic information starting to be used for dose setting and to understand internal dose, e.g., via dose proportionality ▪Microsampling approaches in use to reduce need for additional TK satellite groups ▪Use of PBPK models starting to be explored to predict internal human exposures ▪Limited use of QSARs, e.g., for read-across ▪Biomonitoring data available for some occupational exposures, usually post-marketing (useful for read-across)	▪Use of preliminary study with fewer animals ▪No obligation to generate kinetic data, e.g., for REACH ▪Difficult to generate kinetic data for UVCBs ▪Biomonitoring data being generated on selected chemicals in the general population ▪Limited use of QSARs or read-across	▪Kinetic data generally not generated ▪Limited use of QSARs ,e.g., for read-across
Opportunities and recommendations	▪Pharmaceuticals are evaluated by a risk-assessment and not by a hazard assessment ▪Dose level at the NOAEL in rodents and non-rodents can be used for the calculation of the first dose in human healthy volunteers ▪Use of MABEL approach for the calculation of the first dose in humans for high risk molecules ▪Exposure at the predicted therapeutic human dose can be used as justification for the dose levels in first toxicological studies ▪Comparative metabolism and kinetic data can be used to select the most appropriate species	▪Use of the minimally toxic dose for chronic and carcinogenicity studies is well accepted for classification purposes and should be extended to shorter term studies i.e., a top dose level based on no more than a 10% decrease in weight gain over the duration of treatment may be considered adequate ▪In addition to “toxicities”, dose level selection should be based on an understanding of TK/ADME ▪Dose levels should be guided by a knowledge or a prediction of human dietary exposure using a margin of exposure approach rather selecting maximum tolerable dose levels (i.e., test multiple of predicted human exposure levels)	▪By using toxicokinetics for UVCBs it may be able to generate kinetic data for constituents of concern only, to inform dose selection	▪A risk-based approach should be considered—a hazard-based approach is likely to “identify” hazards that are often many orders or magnitude in excess of any possibility of human exposure ▪It can normally be demonstrated that humans are exposed to very low doses ▪Often very low doses can be used to meet the scientific objectives and application of standard uncertainty factors ▪The entire body of information should be considered before determining the doses to be administered in repeated dose toxicology studies (e.g., in silico methodology, in vitro studies, range finding studies, TK data, read-across)

Updated from Table 1 in Sewell et al 2017 and Table 3 in ECETOC Report 138, 2021

*ADME* absorption, distribution, metabolism and excretion, *CLP* classification labelling and packaging, *DART* developmental and reproductive toxicology, *EPA* *US* environmental protection agency, *HNSTD* highest non-severely toxic dose, *ICH* the international council for harmonisation of technical requirements for pharmaceuticals for human use, *MABEL* minimal anticipated biological effect level, *MOA* mechanism of action, *NOAEL* no observed adverse effect level, *OECD* organisation for economic co-operation and development, *PBPK* physiological based pharmacokinetic, *REACH* registration evaluation authorisation and restriction of chemicals, *STD10* severely toxic dose in 10% of the animals, *TG* test guideline, *TK* toxicokinetics, *TTC* threshold of toxicological concern, *UVCBs* substances of unknown or variable composition complex reaction or biological materials, *QSAR* quantitative structure–activity relationship

### Use of kinetics to inform dose selection

Advice in OECD Guidance Document 116 (OECD 2014) encourages registrants to gain information on systemic exposure (i.e., internal dose) through the use of kinetics as a key part of dose level selection.

*‘…Criteria that have evolved for the selection of an adequate top dose level include: (in particular) toxicokinetics; saturation of absorption…. Toxicokinetic non-linearity should also be considered in the selection of the top dose to be used. Although top dose selection based on identification of inflection points in toxicokinetic non-linearity may result in study designs that fail to identify traditional target organ or body weight effects, it must be appreciated that metabolic saturation in fact represents an equivalent indicator of biological stress. In this case, the stress is evidenced by appearance of non-linear toxicokinetics rather than appearance of histological damage, adverse changes in clinical chemistry, haematology parameters or decrease in body weight gain…’.*

Inclusion of kinetics (pharmacokinetics, toxicokinetics) in regulatory guidance has been developing across all regulated industry sectors over the last few decades but there is ongoing debate on the opportunities that integration of kinetics provides for dose level selection. Therefore, there are variable levels of implementation and regulatory confidence across sectors for both industry registrants and the receiving authorities.

In general, kinetic data can be used to develop a better understanding of the systemic toxicity observed (i.e., to better link systemic exposure levels to observed toxicities), and indeed the combination of the two pieces of information can be extremely useful to inform the design of toxicity studies. Systemic exposure to parent compound and major, measurable and/or relevant toxic metabolites may change with increasing dose, sex, duration, route of exposure, etc. When kinetic data are used in the context of dose selection, the first question to be addressed is whether the substance is absorbed via the relevant route and to determine the relationship between the administered/targeted dose (“external”) and the systemic exposure (“internal”). If that relationship is dose-proportional, doubling the administered dose doubles the systemic exposure. However, a non-dose proportional relationship may result from saturable processes in the ADME of the substance so that levels that reach the systemic circulation do not reflect changes to external/administered dose. This applies to both the parent compound as well as its metabolites.

For animal data, a translational understanding of human relevance of kinetics is important to inform study design in relation to several scopes, including hazard identification, risk assessment, and first dose in human (for pharmaceuticals). Typically, the risk of non-relevance may be higher at higher dose levels, where: A) high systemic exposures may disrupt physiological detoxification processes or other homeostatic processes leading to overt toxicity, potentially confounding appropriate evaluation of the toxicological results, and B) high systemic exposures may be quantitatively and qualitatively different from potential human systemic exposure. Therefore, both aspects may impact the relevance of the observed high dose effects, for human safety hazard identification and risk assessment, with implication on animal use and welfare.

**Fig. 1:**
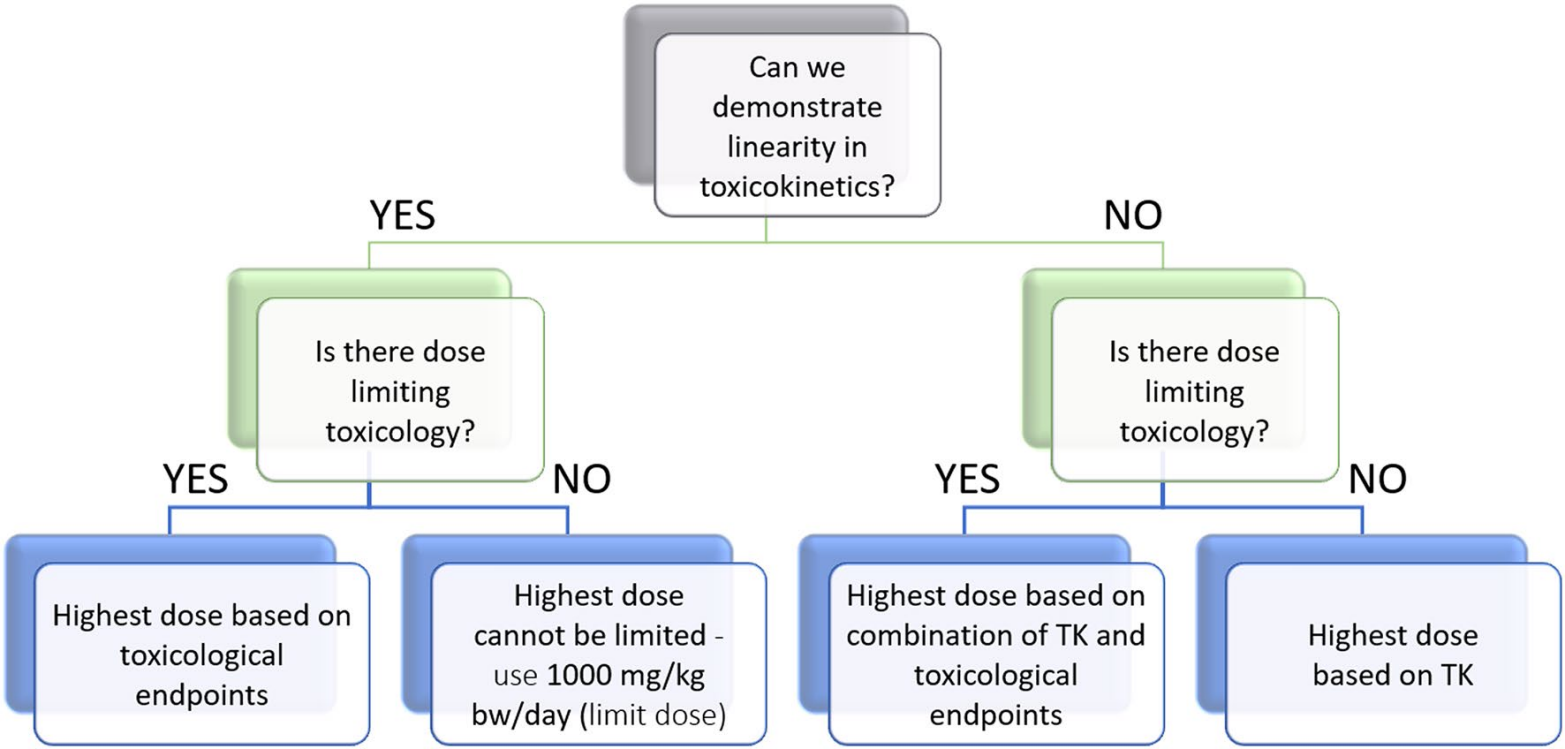
Use of TK data in the design of toxicity studies. The decision flowchart depicts to what extent and in which cases TK information can be integrated in the selection of the high dose for toxicity studies

Kinetic understanding can be obtained using a variety of tools (including in silico*, *in vitro and in vivo), and subsequently used to inform and improve the dose level selection process (Fig. 1; Adapted from ECHA 2017). A weight of evidence approach, incorporating TK information, can be used to set the highest dose level in chronic studies, to consider the dose-proportionality range in systemic exposure without reaching the toxicity MTD based on apical endpoints (such as clinical signs, body weight losses, reduction in body weight gains or organ toxicity). If there is non-linearity in TK and no dose-limiting toxicity, then the high dose can be selected based on TK data. This concept is illustrated in Fig. 1.

The integration of TK information uses relevant departures from dose-proportionality in one or more biomarkers (i.e., parent compound and/or major metabolites) to inform dose level selection in toxicology studies. Thus, TK information can be used as part of the weight of evidence to determine at what dose level systemic exposures become *non-dose proportional* (i.e., due to saturation of metabolic or excretion processes), providing a scientifically-defensible biological basis for selection of lower doses than might otherwise be used in conventional MTD-based testing. The weight of evidence approach is conducted considering kinetic information along with apical endpoint data and estimated human exposures are utilized to scientifically derive a high dose in a longer-term toxicity study. There is limited value in administering doses at levels where increases correspond to minimal increases in systemic exposure (such as the cases of absorption saturation) or, vice versa, where the dose increase may lead to exaggerated systemic exposure not compatible with life over longer exposure durations inducing unnecessary suffering in animals.

**Fig. 2:**
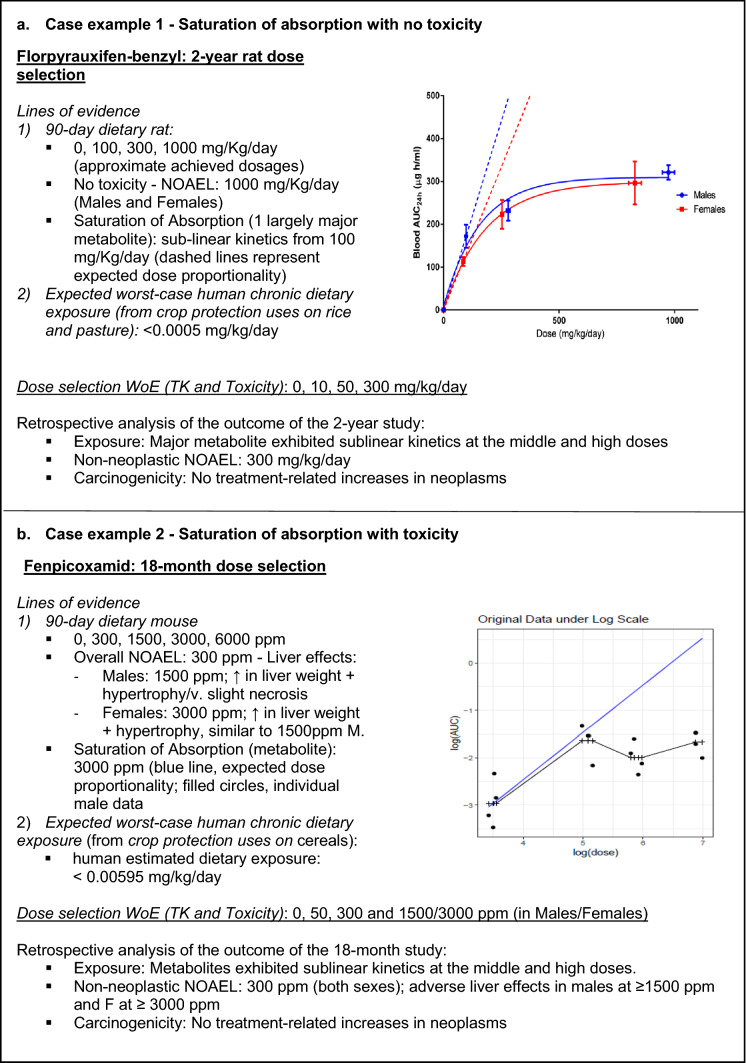
Examples of integration of pharmacokinetics (PK) into dose selection according to the proposed flowchart (Fig. 1). Graphs represent data from 90-day studies. In Case example 1, the group mean and standard error are depicted; dashed lines represent projection of theoretical dose proportional AUCs; in Case example 2, individual male animal data are depicted (filled circles); the blue line depicts the theoretical projection of dose proportionality based on the mean value. In both graphs, dose is test material intake in mg/kg/day

Representative examples of dose selection rationale integrating kinetic information are provided in Fig. 2. In the first case example (Fig. 2 panel A) no toxicity was observed at or below the limit dose (1000 mg/kg/day) in the 90-day rat study with florpyrauxifen benzyl. In addition, the systemic exposure metric of blood area under the curve (AUC) of florpyrauxifen acid (the major metabolite, largely representing systemic exposure) indicated saturation of absorption at doses greater than 100 mg/kg/day for both sexes. Therefore, in this case, TK was used to set the high dose level at 300 mg/kg/day in the 2-year rat study. In another example with fenpicoxamid (Fig. 2, Panel B), toxicity was observed in male and female mice in a 90-day study. With increasing dose levels, TK data indicated that saturation of absorption occurred in males and females at 3000 ppm. Based on apical endpoint toxicity and TK data, concentration of metabolite in plasma, the doses selected for the mouse oncogenicity study were dietary concentrations of 50, 300, 1500 (males) and 3000 (females). It is recommended that evaluation of target organ toxicity is performed in a dose range covering dose proportional TK, thus avoiding use of high doses in the non-dose proportional range.

Integration of TK, the so-called ‘kinetically defined maximum dose’ (KMD) approach, has generated some attention and discussion in recent years, and is not intended to represent the “point of inflection” in a dose-proportionality curve (Slob et al. 2020), nor a blanket approach to be applied in all cases. It is intended as an approach where TK data is used to determine at what dose level systemic exposures become *significantly non-dose proportional* (i.e., due to the contribution of many saturable and non-saturable processes involved, such as metabolism or elimination processes), providing a scientifically-defensible biological basis to inform dose selection, allowing selection of lower doses than might otherwise be used in conventional MTD-based testing (Sewell et al. 2020; Terry et al. 2020; Smith and Perfetti 2020). It is clear that a solid weight of evidence approach should be used, where identification of non-linearities in individual kinetic processes can help identify ranges of non-dose proportionality and, these need to be considered in parallel to toxicity features (Terry et al. 2015). More recently, two dedicated virtual meetings on the selection of doses for long term repeat dose studies have occurred: a plenary session (co-sponsored by U.S. Environmental Protection Agency Office of Pesticide Programs (EPA-OPP), the National Toxicology Program (NTP) Interagency Center for the Evaluation of Alternative Toxicological Methods (NICEATM), and Health and Environmental Sciences Institute (HESI), and a symposium on dose level setting at the US Society of Toxicology (SOT) meeting in 2021, as well as sessions at the more recent SOT meeting in 2022. This work has been driven by the HESI PBPK committee, a consortium of government/regulatory agencies, non-profit organisations, academia, consultants, and industry, which provides a platform for discussion of challenges relating to PBPK. Current focus includes the regulatory use of PBPK and guidance in the use of TK in repeat dose studies to set dose levels and aid interpretation of dose response study results for chemical risk assessment. Progress has been made in identifying barriers and providing recommendations on the definition of the inflection point, human exposure prediction, 3Rs aspects, pros and cons of integrating TK, and how to integrate the various lines of evidence into an organized weight of evidence framework (Tan et al. 2021). More areas of development include statistical and PBPK modelling approaches to determine non-linearity in dose response, and the use of new approach methodologies (NAMs) to support dose selection. PBPK modelling can predict external-systemic dose response relationships, essential for study design and data interpretation. One of the potential desired outputs is to inform dose setting using in vitro kinetic data and sparse in vivo concentration–time data. Conduct of statistical analysis to inform best practices (e.g., selection of number of dose groups and number of animals) is also being investigated. Finally, international discussion has been established between the HESI committee and the OECD working group of OECD National coordinators of the test guidelines programme (WNT) for further scientific exchange in the area of integration of kinetics evidence in toxicity testing (Sewell and Domoradzki, personal communication).

#### Collecting and analysing relevant kinetic information

Developing robust kinetic data useful for dose selection requires an understanding of the underpinning mechanisms of ADME, a selection of suitable “markers” of exposure (analytes to be followed in kinetics/integrated kinetics studies in several matrices, usually blood/plasma and/or urine) and related modelling capabilities.

The ECETOC guidance proposes “modules” where the use of several tools is pragmatically explained for generating information sufficient for dose level selection. Certain sets of information (such as in vitro and PBPK models) are critical in sectors where animals cannot be used (i.e., consumer products) and indeed to also build knowledge of human kinetics where human testing is not permitted. In other sectors the possibility of integrating kinetics into in vivo studies, without using additional animals with the use of microsampling is certainly a powerful tool to investigate systemic exposure across sex, life stages, duration of treatment and dosing condition (Chapman et al. 2014). The availability of radiolabelled compound, analytical standards and analytical capabilities may vary from sector to sector and in different research and development stages. At early stages of a toxicological program, several flexible designs and approaches can be used to obtain first indications of whether the parent and/or metabolites show non-dose proportionality. Once a clear understanding of the main absorption, metabolism and excretion are characterized with sufficient confidence, a selection of metabolites in a representative biological matrix (usually blood/plasma or urine) is then followed in integrated or bespoke kinetic studies, to understand systemic exposure. The ECETOC guidance outlines *study objectives* for integrated kinetics as to:

- Assess dose proportionality of parent compound (and/or relevant toxophores, such as metabolites) under steady state conditions
- Establish gender effects (differences in systemic exposure (AUC) between the sexes)
- Establish temporal effects (changes in systemic exposure (AUC) appearing with time that cannot be explained from single dose data)

## Basic cross-sector information that should be used to guide dose level selection

Different industry sectors have different levels of freedom to operate in relation to dose level selection for repeat dose studies. However, there is some basic knowledge that should be considered prior to repeat dose studies regardless of the industry sector. A review of existing information is important for decisions such as the route of administration, the choice of dosing vehicle, the selection of animal species, and dosages and potential modifications of the dosing schedule. Therefore, all relevant available information on the test material, including physico-chemical, TK (including species-specific metabolism), toxicodynamic properties, structure–activity relationships (SARs), and in vitro metabolic processes should be considered. Limited predictions of ADME and bioaccumulation may be derived from chemical structure, physico-chemical data, and information on the extent of plasma protein binding or from TK studies, while results from existing toxicity studies will give additional information, e.g., on NOAEL, metabolism or induction of metabolism (OECD TG 443, 2018).

Before moving on to selecting appropriate dose levels, the investigator should consider all available information, including (but not limited to):

- The plethora of large data bases of in vivo toxicity and computer-based models can be used to obtain an understanding of chemical structures and mechanisms, facilitating focus on the key toxicological endpoints that should be considered. For example, databases that are searchable by chemical structure, such as the US EPA’s CompTox Dashboard, can be used along with read across approaches to find acceptable analogs which already have toxicity data, negating the need for testing. In some circumstances, if a specific read-across candidate is read across along with Quantitative Structure Activity Relationships (QSARs) this may highlight data for substances that are partially similar, e.g., through the sharing of functional groups, and provide understanding of possible modes of action, target organs and indications of data which may help in selecting dose groups for a target substance. The most easily accessible and widely used tool is the OECD QSAR toolbox (OECD 2021). Use of this software can identify structurally similar substances and identify opportunities for read-across, and the profiling module is also a good early warning system to indicate toxicological mechanisms or endpoints that could be sensitive during in vivo testing.
- Results of existing toxicity studies, including known hazardous properties such as extensive irritation which can cause a disruption in the natural barrier of the tissue system at the portal of entry (whether the skin, the gastric lining or the nasal epithelium), with inflammation, hyperkeratosis, ulceration and breakdown of epithelial integrity (cytotoxicity, venous access, scarring etc.). This can result in altered absorption of the material and unrealistic dosing and exposure scenarios. It is equally important to consider any animal welfare concerns of testing such chemicals. Approaches should be taken to minimise potential pain and distress, which can be challenging to determine in laboratory animals.
- A knowledge of TK as described earlier.
- A knowledge of sector, foreseeable conditions and exposure scenarios.

## Conclusions and recommendations

The recommendations of the ECETOC report represent pragmatic approaches to selecting dose levels that allow for accurate risk assessment but also enable hazard-based classification based on identification of relevant hazards and take into account sector-specific differences. These can be applied within the current regulatory frameworks, but also cover some forward-looking future options and approaches to dose level selection.

As recommended in test guidelines and guidance documents, wherever practically possible an understanding of systemic exposure should be developed (e.g., through the deployment of TK approaches) and used to guide dose level selection. Though in the majority of cases systemic exposure (blood and tissue) will be linear with applied external dose, demonstrating this provides reassurance that the biological effects (including the toxicities that are observed), represent true responses to increasing exposure. In a minority of cases a less than proportional increase in systemic exposure may be demonstrated. In such a situation this knowledge is vital in shaping approaches to dose level selection where plateaus of exposure or less than proportional exposure with increasing applied dose can be taken into account. This information must come from appropriate and rigorous TK approaches.

Where there are no or little data to make a dose selection decision based on systemic exposure, or where systemic exposure has a linear relationship with the externally applied/targeted dose, then signs of toxicity remain the main source of knowledge for selecting appropriate dose levels. With the possible exception of early dose range-finding studies and in the absence of any clear prior information on mode of action or structural class to guide dose level selection, the highest dose level in repeat dose studies should be limited to a reasonable level such as that which causes evident but minimal toxicity (as an example, existing guidance often points to effects such as a 10% reduction in body weight gain). There is no scientific justification/value in selecting the high dose in repeat dose studies with the aim of causing overt/significant systemic toxicity (i.e., pain, distress, suffering) or lethality. In practice, laboratories and investigators conducting studies have very clear local guidance and legislation aimed to limit or prevent the use of doses that cause such effects. As an example, any repeat dose study causing lethality, peri-lethal effects or a sustained period of reduced weight gain or weight loss at the high dose would very likely be terminated without further evaluation and further studies conducted at more appropriate dose levels.

As the science of predictive human exposure further develops and matures, this will provide exciting and novel opportunities for more relevant approaches to dose level selection. In some sectors (e.g., pharmaceuticals) this approach is well understood and is currently used in dose level selection; in other sectors such as agrochemicals, the knowledge and understanding needed to support a margin of exposure approach is developing.

In circumstances where the mode of (toxic) action is well understood and described, and where a material can be clearly assigned to such a class, then different opportunities exist and should be considered in approaches to dose level selection. It is, however, recognised that to base doses on the mechanism of action, one would need a quite extensive and existing knowledge base. Similarly, the paradigm for dose level selection in studies designed to elucidate a mechanism of action (usually based on the findings from more traditional regulatory toxicity studies), can be very different and need to be designed on a case-by-case basis.

## Abbreviations

3Rs: Replacement, reduction and refinement of animals in research
AUC: Area under the curve
CLP: Classification labelling and packaging
CMR: Carcinogenicity, mutagenicity and reproductive toxicity
DNELs: Derived no effect levels
ECETOC: European centre for ecotoxicology and toxicology of chemicals
GHS: Globally harmonised system
HESI: Health and environmental sciences institute
ILSI: International life sciences institute
MTD: Maximum tolerated dose
NOAEL: No observed adverse effect level
PBPK: Physiologically based pharmacokinetic
PK: Pharmacokinetic
PoD: Point of departure
ppm: Part per million
PNECs: Predicted no effect concentrations
